# *In Vitro* and *In Vivo* Antifungal Activity of Lichochalcone-A against *Candida albicans* Biofilms

**DOI:** 10.1371/journal.pone.0157188

**Published:** 2016-06-10

**Authors:** Dalia Seleem, Bruna Benso, Juliana Noguti, Vanessa Pardi, Ramiro Mendonça Murata

**Affiliations:** 1 Herman Ostrow School of Dentistry, Division of Periodontology Diagnostic Sciences, Dental Hygiene and Biomedical Sciences, University of Southern California, Los Angeles, CA, United States of America; 2 School of Dentistry, Faculty of Medicine, Universidad Austral de Chile, Campus Isla Teja, Valdivia, Chile; Yonsei University, REPUBLIC OF KOREA

## Abstract

Oral candidiasis (OC) is an opportunistic fungal infection with high prevalence among immunocompromised patients. *Candida albicans* is the most common fungal pathogen responsible for OC, often manifested in denture stomatitis and oral thrush. Virulence factors, such as biofilms formation and secretion of proteolytic enzymes, are key components in the pathogenicity of *C*. *albicans*. Given the limited number of available antifungal therapies and the increase in antifungal resistance, demand the search for new safe and effective antifungal treatments. Lichochalcone-A is a polyphenol natural compound, known for its broad protective activities, as an antimicrobial agent. In this study, we investigated the antifungal activity of lichochalcone-A against *C*. *albicans* biofilms both *in vitro* and *in vivo*. Lichochalcone-A (625 μM; equivalent to 10x MIC) significantly reduced *C*. *albicans* (MYA 2876) biofilm growth compared to the vehicle control group (1% ethanol), as indicated by the reduction in the colony formation unit (CFU)/ml/g of biofilm dry weight. Furthermore, proteolytic enzymatic activities of proteinases and phospholipases, secreted by *C*. *albicans* were significantly decreased in the lichochalcone-A treated biofilms. *In vivo* model utilized longitudinal imaging of OC fungal load using a bioluminescent-engineered *C*. *albicans* (*SKCa23-ActgLUC)* and coelenterazine substrate. Mice treated with lichochalcone-A topical treatments exhibited a significant reduction in total photon flux over 4 and 5 days post-infection. Similarly, *ex vivo* analysis of tongue samples, showed a significant decrease in CFU/ml/mg in tongue tissue sample of lichochalcone-A treated group, which suggest the potential of lichochalcone-A as a novel antifungal agent for future clinical use.

## Introduction

Oral candidiasis (OC) is one of the most common fungal infections affecting the oral cavity [1]. *Candida albicans*, is a prevalent opportunistic human fungal pathogen that is often implicated in OC. *C*. *albicans* lives commensally in the gut, oral pharyngeal, genito-urinary tract and skin [2]. However, pathogenicity and subsequent candidiasis can occur under immunocompromised conditions [3,4]. For instance, the incidence of at least one episode of oral candidiasis in HIV patients is estimated to be 80–95% [5]. As a consequence of oral fungal infections, patients may have dysphagia, weight loss, or disseminated candidiasis. The disseminated forms of the disease can be life-threatening with mortality rates of 35–60% among immunocompromised, cancer patients, or those exposed to multiple treatments, such as broad spectrum antibiotics, chemotherapy, immunosuppressive therapy, and anti-retroviral therapy [6–8]. The pathogenicity of the *Candida* species is attributed to critical virulence factors, such as evasion of host defenses, adherence to surfaces (on both tissues and medical devices), biofilm formation, and production of proteolytic enzymes, such as secreted aspartyl proteases (SAP) and phospholipases [9].

Biofilm formation is considered a critical virulence factor of *C*. *albicans* that distinguishes it from its free- floating or planktonic counterpart and contributes to its antifungal resistance [10]. Biofilm development tends to occur in 4 sequential steps; first, adhesion of a microorganism to a surface, followed by initiation of hyphal growth. Then, more extracellular matrix is accumulated in the maturation step and the biofilm structure is formed. Finally, yeast cells detach and invade surrounding tissues. Mature fungal biofilms are characterized by a dense community of both yeasts and hyphae encased in a thick extracellular polymeric substance (EPS), which ensures adequate diet is supplied to biofilms, transports waste products, and may also have a role in the antifungal resistance of *Candida* species [11]. In addition, hyphae formation is considered the most critical factor in inducing epithelial invasion, which triggers the degradation of epithelial cell junction proteins [12].

Another virulence factor associated with the pathogenicity of *C*. *albicans* is secretion of proteolytic enzymes, such as secreted aspartyl proteases (SAP) and phospholipases [3,9,13]. SAPs have been reported to elicit a destructive effect on the host tissue during mucosal infections, as they facilitate hyphal invasion and activate the degradation of E-cadherin, a major protein present in epithelial cell junction [12,13].

Despite the availability of broad spectrum triazoles as conventional medical therapies, the incidence of invasive candidiasis continue to increase due to the antifungal resistance of *Candida* species to such antifungal agents [14]. Thus, there is an urgent need to evaluate novel compounds with antifungal activity. Flavonoids are a major class of natural compounds known as polyphenols, which are secondary metabolites naturally occurring in plants and found largely in foods and beverages, such as fruits, vegetables, cereals, tea, coffee, and red wine [15,16]. Lichochalcone-A is a bioactive natural compound found in licorice roots of *Glycyrrhiza* species, which has been used as a traditional herbal remedy [17]. Licorice contains several classes of secondary metabolites with which numerous human health benefits have been associated. Recent research suggested that licochalcone-A possesses potential beneficial effects against oral diseases, such as periodontitis, candidiasis, and recurrent aphthous ulcers [17]. In one study, lichochalcone-A has been shown to have antimicrobial effects, as it inhibited biofilm formation in *Streptococcus suis* as well as suilysin secretion [18].

The aims of the present study were to evaluate the antifungal activity of lichochalcone-A against *C*. *albicans in vitro* and to determine if lichochalcone-A can disrupt biofilm formation by reducing critical virulence factors associated with *C*. *albicans*, such as secretion of proteolytic enzymes, which are often implicated in the degradation of host mucosal tissue [3]. Furthermore, a novel *in vivo* bioluminescent mouse model of OC was studied to investigate real-time progression of the fungal infection [19] as well as the effectiveness of topical treatments of lichochalcone-A. The significance of this study is to validate the antifungal activity as well as the safety of lichochalcone-A use *in vivo* to treat and/or prevent oral candidiasis.

## Materials and Methods

### Test agents

**Fig 1:**
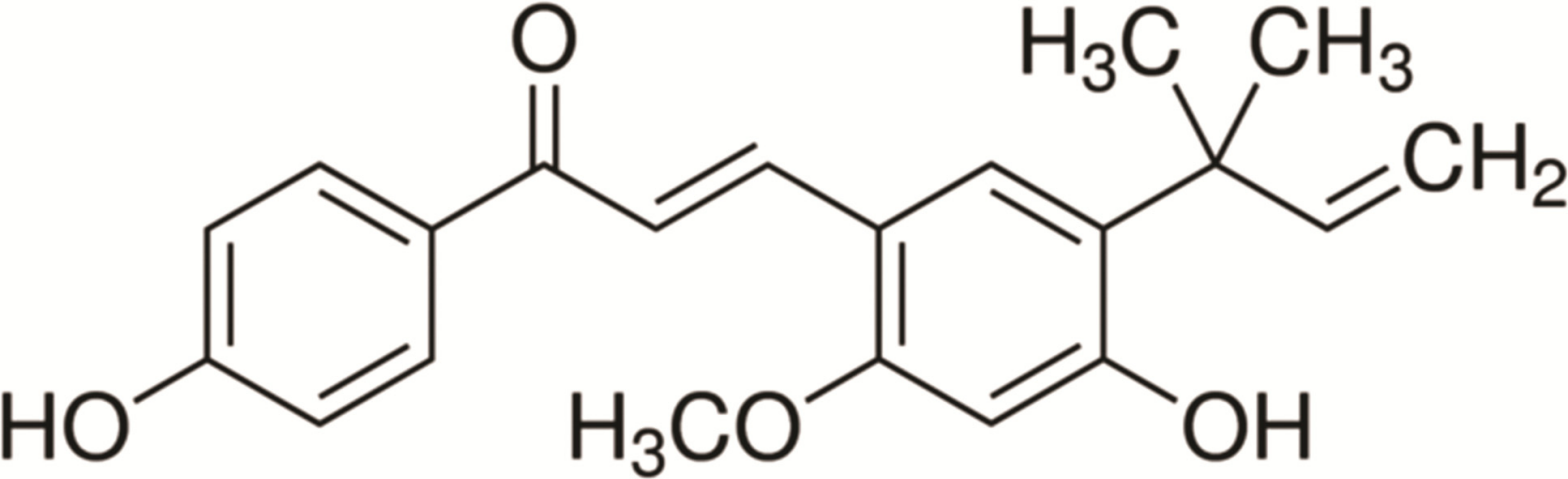
Structure of lichochalcone-A.

Pure extract of lichochalcone-A (99.9% high-performance liquid chromatography grade) (ALX-430-124-M005) was purchased from sigma- Aldrich. The structure of lichochalcone-A is shown in Fig 1. A stock solution of lichochalcone-A was prepared at 28 mM in 100% (v/v) ethanol. Serial dilutions of lichochalcone-A (2.8–280 μM) were prepared at a final concentration of 1% ethanol. Fluconazole (32–320 μM) (Sigma) and nystatin (100 mM) served as positive controls and 1% (v/v) ethanol was the vehicle control used in the experiments. All solutions were prepared fresh.

### Microorganisms

*In vitro* assays were conducted using *C*. *albicans* strains MYA2876, ATCC 90028, and a fluconazole-resistant *C*. *albicans* strain 321182, which were selected as proven virulent pathogens with known genomic sequences. *In vivo* experiments were performed on *SKCa23-ActgLUC*, which is an engineered Wild-type *C*. *albicans* SC5314, to express *C*. *albicans* codon-optimized *Gaussia princeps* luciferase (*gLuc*) fused to the endogenous *PGA59* gene at the cell wall, under the control of a constitutive (actin, *ACT1*) and a hyphal growth phase-specific (*HWP1*) promoter [20]. These strains were kindly provided to us by Dr. Van Dijck (VIB, Leuven, Belgium).

### Susceptibility Test

The antimicrobial activity of lichochalcone-A (Sigma) was tested *in vitro* against the following *C*. *albicans* strains: MYA2876, ATCC 90028, *SKCa23-ActgLUC*, and resistant strain 321182, following the NCCLS guidelines, as outlined by [21]. The minimum inhibitory concentration (MIC) was determined using an inoculum of 5x10^3^ CFU/ml *C*. *albicans* grown in RPMI-1640 (Lonza) in 96 well-plates incubated with 10% final volume of the tested compound or the controls. Inoculum concentration was standardized using a spectrophotomer, by first measuring the absorbance in the range of 0.08–0.1 at 625 nm, which yielded a yeast stock solution equivalent to 5x10^6^ CFU/ml that was then diluted to a final ratio of 1:2000, to result in 5x10^3^ CFU/ml. For the tested compound, serial dilutions in the range of 2.8–280 μM of lichochalcone-A (Sigma) were prepared. Fluconazole (10–1000 μM) (Sigma) was tested to establish the MIC specific for each strain, which served as a positive control for the susceptibility assay of the tested compound. The vehicle control used was 1% Ethanol (v/v). All plates were incubated for 24 hours at 37°C in 5% CO_2_. Minimum inhibitory concentration (MIC) was determined after 24 hours as the concentration at which *C*. *albicans* growth was visibly inhibited [22,23]. Minimum fungicidal concentration (MFC) was found by subculturing 20 μl of each well that had concentrations above the MIC on Sabouraud Dextrose Agar (BD) after 24 hours of incubation at 37°C in 5% CO_2_. The MFC concentration was determined as the lowest concentration of lichochalcone-A showing no visible *C*. *albicans* growth on the agar plates. All of the assays were performed in triplicates and repeated at least three different times for reproducibility.

### Time-kill assay

Initial *C*. *albicans* (MYA2876) inoculum was prepared in 10³ CFU/ml in RPMI 1640 medium using spectrophotometric methods according to NCCLS protocol (2002), as previously described [21]. Lichochalcone-A at concentrations equal to 10 and 20 times the MIC for the tested isolate were added to the inoculum concentrations in 96-well plates, as 10% of total solution volume in each well. Other tested groups included fluconazole (32 μM) (positive control), 1% ethanol (vehicle control), and growth medium with inoculum only. Test solutions were placed on a shaker and incubated at 35°C. At predetermined time points (0, 1, 2, 4, and 24 h) following the introduction of the test isolate into the system, 100 μl aliquots were removed from each test solution. Tenfold serial dilutions were performed on all samples, and a 10 μl aliquot from each dilution was plated on a sabouraud dextrose agar plate for colony count determination. These procedures were performed to eliminate any potential antifungal carryover effect [24]. Following incubation at 35°C for 48 h, the number of CFU on each plate was determined. Time-kill assay experiments were performed in triplicates and repeated on three different occasions.

### Biofilm Assay

An inoculum of 1x10^6^ CFU/ml of *C*. *albicans* (MYA 2876) was formed in a sterile 24-well plate using Yeast Nitrogen Base Medium (Difco) with 50 mM of glucose and incubated at 37°C in 5% CO_2_ for 24 hours to establish initial biofilm growth, as performed by [25]. Total volume of 1 ml of inoculum was pipetted in each well. After 24 hours of incubation, the biofilms were treated twice daily with lichochalcone-A at concentrations of 625 μM (equivalent to 10x MIC). The vehicle control used was 1% ethanol while fluconazole (320 μM) was the positive control. Biofilms were washed daily with Phosphate Buffer Solution (PBS) and replenished with fresh medium. After 72 hours of treatments, adhered biofilms were collected by scraping the bottom of each well plate and suspending biofilms in PBS, which was then centrifuged at 10,000 rpm for 5 minutes. Biomass (dry weight) of each biofilm sample was obtained by discarding the supernatant and placing the samples in a speed vacuum to dry for 40 minutes [26]. Colony formation unit (CFU) was determined by suspending each sample of biofilm in 1 ml of PBS and plating 20 μl of the suspension on Sabouraud Dextrose Agar plates (BD), which were incubated at 37°C in 5% CO_2_ [27,28]. After 24 hours of incubation, the number of *C*. *albicans* colonies was counted and the data was normalized based on the colony count/ml/dry weight of biofilm sample.

### Proteinase and Phospholipase Enzyme Secretion Assay

Proteinase and phospholipase enzyme secretion assays were conducted as previously performed by Santana et al., 2013 [26]. Biofilms of *C*. *albicans* were grown for 24 hours in Yeast Nitrogen Base Medium (Difco) with 50 mM of glucose at 37°C in 5% CO_2_ and treated with lichochalcone-A at concentrations of 625 μM and 1500 μM, which were equivalent to 10x the range of MIC (62.5 μM- 150 μM) for the specific strain used in this assay, MYA2876. The vehicle control used was 1% ethanol. After 72 hours of biofilm maturation, the enzyme secretion assays were performed on the sonicated biofilms, suspended in PBS. The proteinase enzyme activity was determined by mixing the supernatant of the biofilm solution with 1% azocasein at 1:9 (v/v) for 1 hour at 37°C in 5% CO_2_. Then, 500 μl of 10% trichloroacetic acid was added to stop the reaction. The solution was centrifuged for 5 minutes at 10,000 rpm and 500 μl of the supernatant was combined with 500 μl of NaOH, which was incubated for 15 minutes at 37°C in 5% CO_2_. Absorbance was read in a spectrophotometer at 440 nm [26,29,30]. The phospholipase enzyme activity was determined by mixing the supernatant of the biofilm solution with phosphatidylcholine substrate for 1 hour at 37°C in 5% CO_2_ followed by reading the absorbance in a spectrophotometer at 630 nm [26,30,31].

### Co-culture Model Fluorescence Microscopy

A co-culture model was conducted by culturing fibroblast cells and *C*. *albicans* together in a sterile 24-well plate, as adapted by [32]. First, oral fibroblast cells (ATCC: CRL2014) were seeded in Dulbecco’s Modified Eagle’s Medium (DMEM) with Fetal Bovine Serum (FBS) (Gibco) at 37°C in 5% CO_2_ for 24 hours. The medium was then replaced with an inoculum of 5x10^3^ to 2.5x10^3^ CFU/ml *C*. *albicans* (MYA2876) grown in DMEM without FBS. Fibroblast cells and *C*. *albicans* were treated with 62.5 μM of lichochalcone-A. The plate was then incubated at 37°C in 5% CO_2_ for 24 hours. The vehicle control tested was 1% ethanol and the positive control was fluconazole (32 μM). The distribution of dead and live fibroblast cells was examined using the viability/cytotoxicity assay kit for animal live & dead cells (Biotium), which contains a mixture of Calcein AM and EthDIII. Calcofluor white (Sigma) was used to stain *C*. *albicans*. Fluorescent images of the double staining were captured using fluorescence microscopy (EVOS fl microscope AMG, Bothell, WA, USA).

### Host inflammatory Cytokines analysis using ELISA

As previously described co-culture models were performed using fibroblasts, *C*. *albicans* (MYA2876), and the tested groups of lichochalcone-A (62.5 μM and 150 μM), positive control (fluconazole 32 μM), and 1% ethanol (vehicle control). After 72 hours of incubation, the supernatants of the biofilms were collected, centrifuged for 10 minutes at 1000 rpm, and assayed immediately using Qiagen single analyte ELISA kit for expression of pro-inflammatory cytokines IL-1α, IL-1β, and anti-inflammatory 1L-10, as described by [33]. Briefly, 96-well ELISArray microplates (Qiagen) were coated with antibodies for IL-1α, IL-1β, and1L-10. Equal volumes of assay buffer (10% BSA) and tested samples were added to the plates, which were incubated for 2 h at 37°C and then washed three times with ELISA wash buffer. Following washing, the plates were incubated with detection antibody solution (100 µl) for 1 h at 37°C, washed 3 times and then incubated with Avidin-HRP for 30 minutes at 37°C. After washing four times, the plates were incubated with development solution (100 μl) in the dark for 15 minutes, followed by the addition of a stop solution (100 µl). The absorbance was read at 450 nm on a SpectraMax M5 Microplate Reader [34]. The results were expressed as average percentages of normalized values based on the vehicle control values set as 100%. All samples were tested in quadruplets at two different occasions.

### Mouse model of oral candidiasis

All protocols and procedures were approved and performed in accordance with the Institutional Animal Care and Use Committee of USC (protocol # 20266), and in accordance with the Panel on Euthanasia of the American Veterinary Medical Association (A3518-0). In this study, 15 male, 6–7 weeks old, inbred *Balb/c* mice were housed in a pathogen-free environment at USC Animal Vivarium under the supervision of full-time veterinarians on call 24 hours/day. To ensure no existing oral fungal infections, the oral cavity of the mice were swabbed with PBS solution and the swabs were spread on Sabouraud Dextrose Agar (BD) plates, which were incubated for 24 hours. Mice were rendered susceptible to oral candidiasis by subcutaneous administration (225 mg/kg) of cortisone acetate (Sigma-Aldrich), which was administered every other day, starting at day 1 relative prior to infection [35]. A 2-day biofilm of *C*. *albicans* (*SKCa23-ActgLUC*) was performed according to the procedures discussed in the *in vitro* biofilm assay and inoculum (1 × 10^7^ cells/ml) was suspended in Hanks’ balanced salt solution (HBSS). On the day of the infection, mice were sedated with isoflurane oral inhalation (2–4%) and the oral cavities were infected with *C*. *albicans* by placing calcium alginate swabs saturated with *C*. *albicans* suspension (1 × 10^7^ cells/ml) sublingually for 75 min. Topical treatments of Lichochalcone-A (7.5 mM, which was equivalent to 100x MIC for *SKCa23-ActgLUC*, dose selected based on prior *in vitro* assays with the strain, data not shown) were applied twice daily starting on day 1 post-infection. The antifungal effect of lichochalcone-A was compared with the positive (nystatin) at 100 mM and the vehicle (1% ethanol) controls (n = 5 in each of the tested groups). Imaging of the infection was performed by pipetting 10 μl of coelenterazine (0.5 mg/ml in1:10 ethanol: PBS) (Promega) substrate into the oral cavities of the mice during sedation with isoflurane oral inhalation cavity and imaging the mice in the IVIS-200TM Imaging system (Xenogen Inc.). Longitudinal imaging of the infection progression was monitored at 1, 4, and 5 days post-infection and the total photon emission from oral areas within the images (region of interest, ROI) of each mouse was quantified with Living ImageR software package [19]. Mice were euthanized on day 6 post-infection via CO_2_ inhalation followed by cervical dislocation. *Ex vivo* analysis of infected tongues were performed by sectioning the tongues for fungal burden and histological evaluation. The fungal burden analysis of the tongue was conducted by plating serial dilutions of organ homogenates onto Sabouraud Dextrose Agar (BD) (Sigma-Aldrich) and normalizing the CFU/ml by the weight of tissue sample (mg).

The histopathological analysis involved fixating the tongues immediately after excision in 10% formalin followed by embedding in paraffin. The tongues were sectioned longitudinally to verify the extension of the lesions, stained using the periodic acid-Schiff (PAS) procedure to visualize fungi, and examined by light microscopy (Leica DM2500). In addition, the *ex vivo* toxicity of lichochalcone-A was investigated through necropsy, gross/macroscopic, and microscopic/histopathologic examination [11].

### Statistical Analysis

The experiments were performed in triplicates and repeated three different times for reproducibility. Data were expressed as means ± SEM and analyzed using JMP software (version Pro 11.0.0; SAS Institute Inc.). Differences between and within the groups were analyzed using parametric or non-parametric measures, as dictated by the results. The level of statistical significance was set at 0.05. Analysis of CFUs reduction were performed using One-way Analysis of Variance (ANOVA), followed by the Dunnett test. To analyze the *in vivo* photon emission quantification, repeated measures of ANOVA was applied (PROC MIXED) using SAS, followed by Tukey-Kramer test (α = 0.05). Results were considered significant if p-values were less than 0.05.

## Results

### *In vitro* antifungal activity

Susceptibility assay of lichochalcone-A against *C*. *albicans* (MYA2876) showed an antifungal activity, as indicated by the minimum inhibitory concentration (MIC) and minimum fungicidal concentration (MFC) values of 62.5–150 μM and 150 μM; respectively, which were comparable to the MIC values of conventional antifungal fluconazole, suggesting similar potency (Table 1) [36]. Other strains were also tested for susceptibility to lichochalcone-A with reported values that were comparable to the positive control, fluconazole. It should be noted that the growth of the fluconazole- resistant strain 321182 was inhibited at a lower concentration of lichochalcone-A compared to that of fluconazole (Table 1).

**Table 1:** Minimum inhibitory concentration (MIC) and minimum fungicidal concentration (MFC) of lichochalcone-A against *Candida albicans*.

Microorganism	Lichochalcone-A		Fluconazole	
	MIC (μM)	MFC (μM)	MIC (μM)	MFC (μM)
*Candida albicans* MYA-2876	62.5–150	150	32	100
*Candida albicans* 90028	62.5–100	100	20	90
*Candida albicans SKCa23-ActgLUC*	50–75	75	10	80
*Candida albicans* 321182	65	150	100	350

*Fluconazole rtesistant.

**Fig 2:**
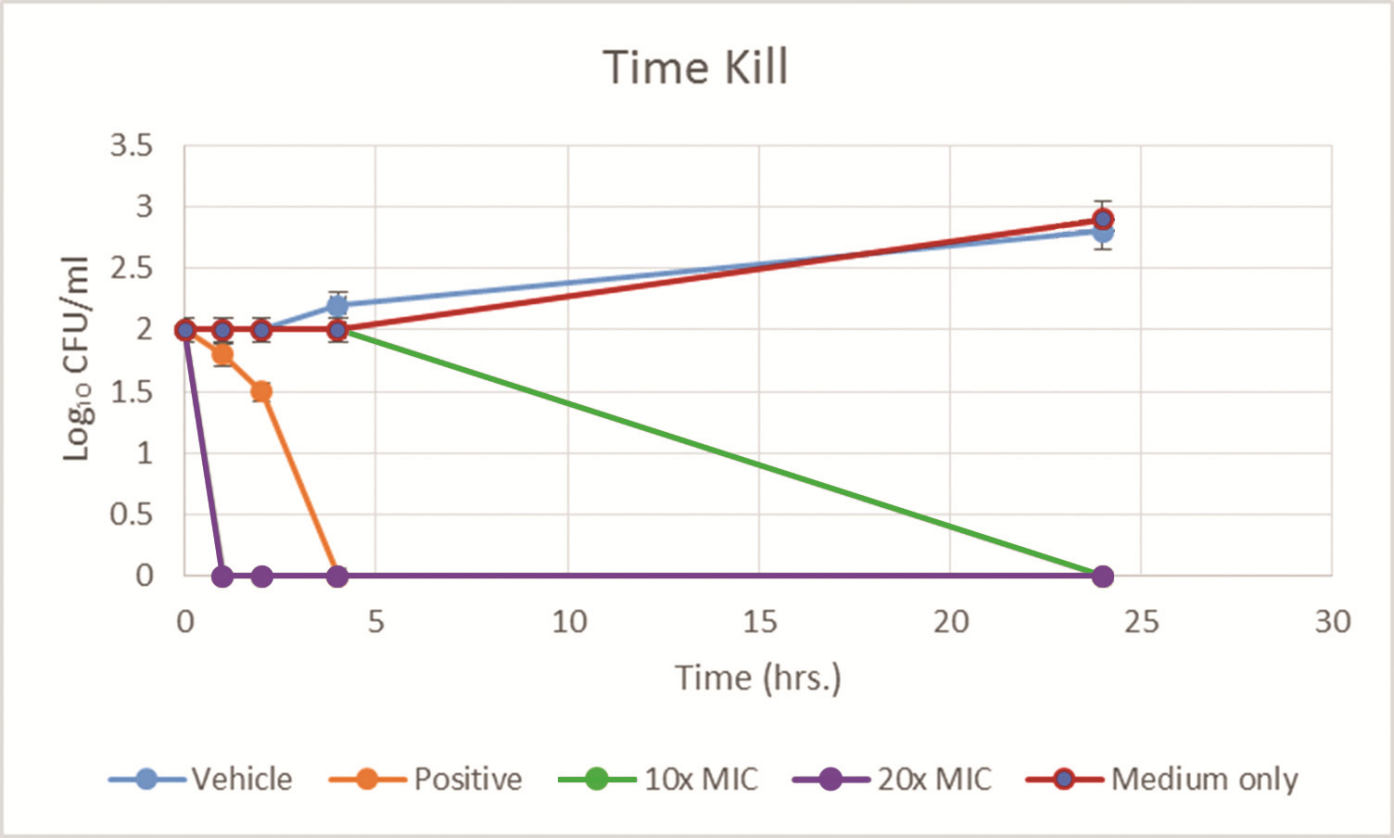
Time Kill of *C*. *albicans* (MYA 2876) inoculum (10³ CFU/ml) tested against lichochalcone-A (at 10x MIC, and 20x MIC), fluconazole 32 μM, (positive control), 1% ethanol (vehicle control), and medium with inoculum only (negative control), plot expressed as average values for log10 of the numbers of CFU/milliliter versus time (hrs.).

Time-kill experiment showed the most decrease in fungal viability occurring in the first 5 hours after application of the lichochalcone-A treatment at 10x MIC (625 μM) and 20x MIC (1250 μM) to *C*. *albicans* (MYA 2876) inoculum (10³ CFU/ml) (Fig 2). At 24 hours, complete fungal load was eradicated, which was suggestive of the potential efficacy of lichochalcone-A as a topical treatment.

### Biofilm inhibition

**Fig 3:**
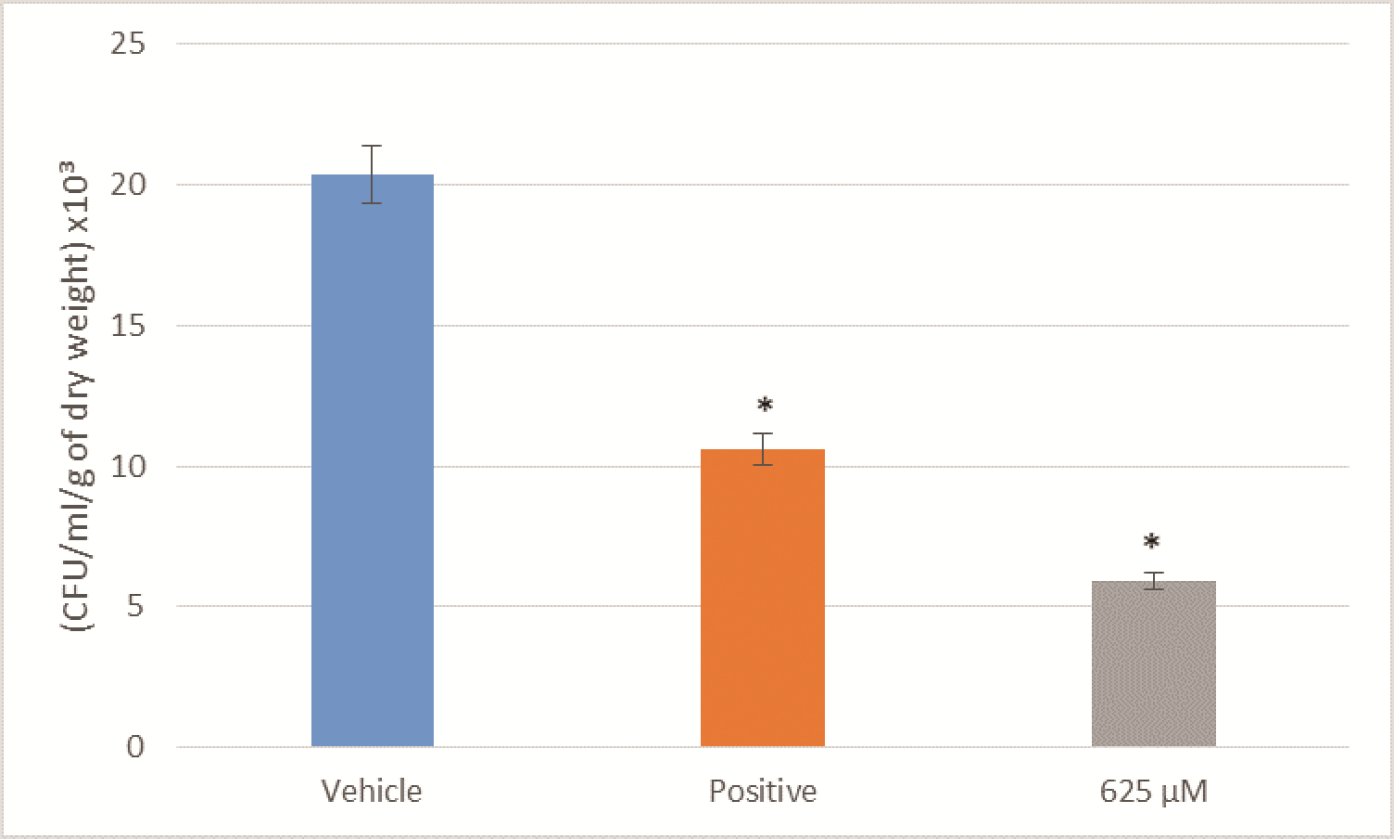
Fungal viability of *C*. *albicans* biofilm expressed in CFU/ml/ grams of dry weight after treatment with lichochalcone-A. The antifungal activity of lichochalcone-A (625 μM; 10x MIC) against *C*. *albicans* MYA 2876 biofilms was compared to the vehicle control group (1% ethanol) and positive control group (fluconazole 320 μM; 10x MIC) The standard deviations of each sample are shown in the graph, and all the mean differences between the control groups and test (lichochalcone-A at 625 μM) were statistically significant (*p<0.05).

Biofilm assay showed significant reduction (p<0.05) in fungal load after treatments with lichochalcone-A at 625 μM (equivalent to 10x MIC for *C*. *albicans* MYA 2876) in comparison to the vehicle control (Fig 3). In addition, the fungal viability of the *C*. *albicans* biofilms treated with lichochalcone-A were lower compared to that of the positive control group, fluconazole at 320 μM concentration, which was equivalent to 10x MIC of fluconazole against MYA 2876. Fungal load was expressed, as colony formation unit (CFU)/ml/ g of biofilm dry weight.

### Co-culture model of fibroblasts and *C*. *albicans*

Oral fibroblasts cells were selected for the co-culture experiments, as they are routinely used to assess the effects of topical antimicrobial application on cell viability [37]. Furthermore, fibroblasts were used in the analysis of host cytokines inflammatory expression, which will be discussed later, as such cells have been shown to have an important role in the oral immune response to *C*. *albicans* infections [38].

**Fig 4:**
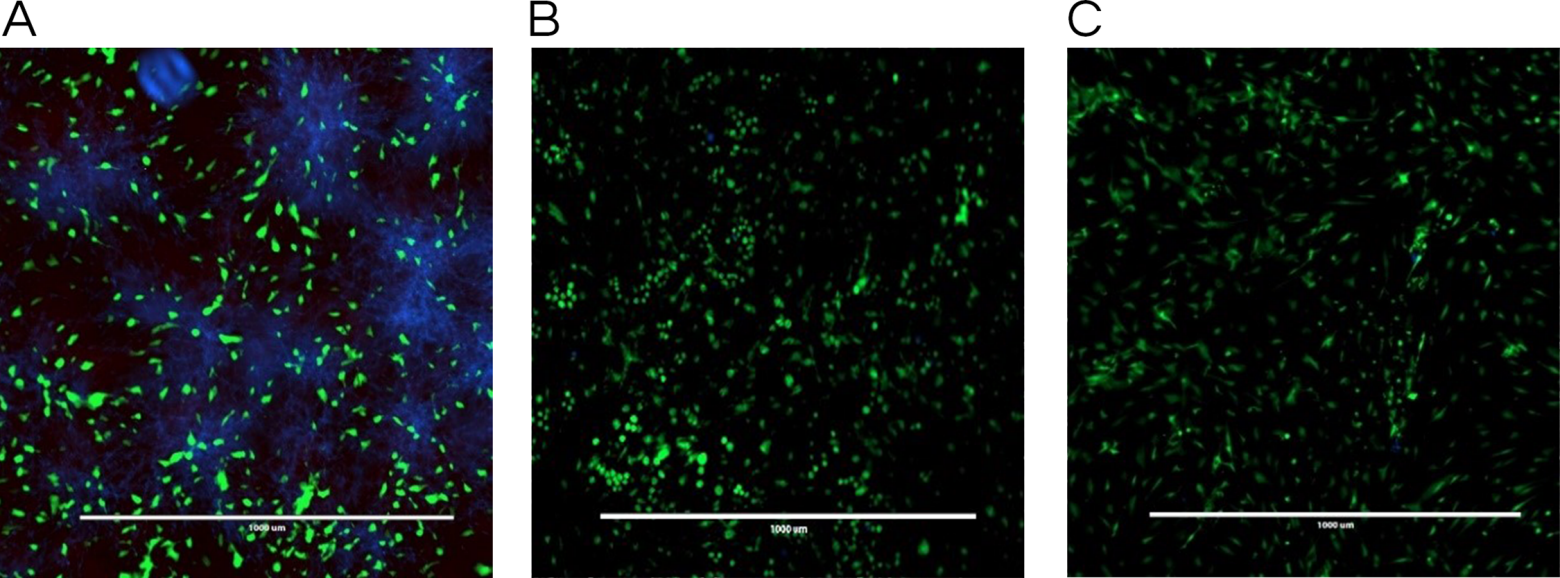
Co-culture fluorescence microscopy stained with calcofluor white stain (Blue: *C*. *albicans*) and Viability/Cytotoxicity Assay Kit for Animal Live & Dead Cells (Green: live fibroblast cells; Red: dead fibroblast cells). **A.** Vehicle control (ethanol 1%); **B.** Positive control (fluconazole 32.2 μM); and **C.** Lichochalcone-A at 62.5 μM. Lichochalcone-A displayed low *candida* (blue fluorescence) growth with even distribution of live fibroblast cells (green fluorescence). Scale bars are set in μm.

In the co-culture model of fibroblasts coexisting with *C*. *albicans*, samples treated with lichochalcone-A showed a considerable decrease in *Candida* growth distribution in comparison with the vehicle control, as indicated by the reduction in the *C*. *albicans* growth (blue color) among fibroblast cells (green color) in fluorescent images (Fig 4). In addition, fibroblasts viability was not adversely affected by the treatment of lichochalcone-A, as there was no increase in the dead fibroblast population, indicated by the red fluorescent color. Thus, lichochalcone-A was effective against *C*. *albicans* with minimal effects or toxicity against fibroblast cells.

### Proteolytic enzymatic activities

**Fig 5:**
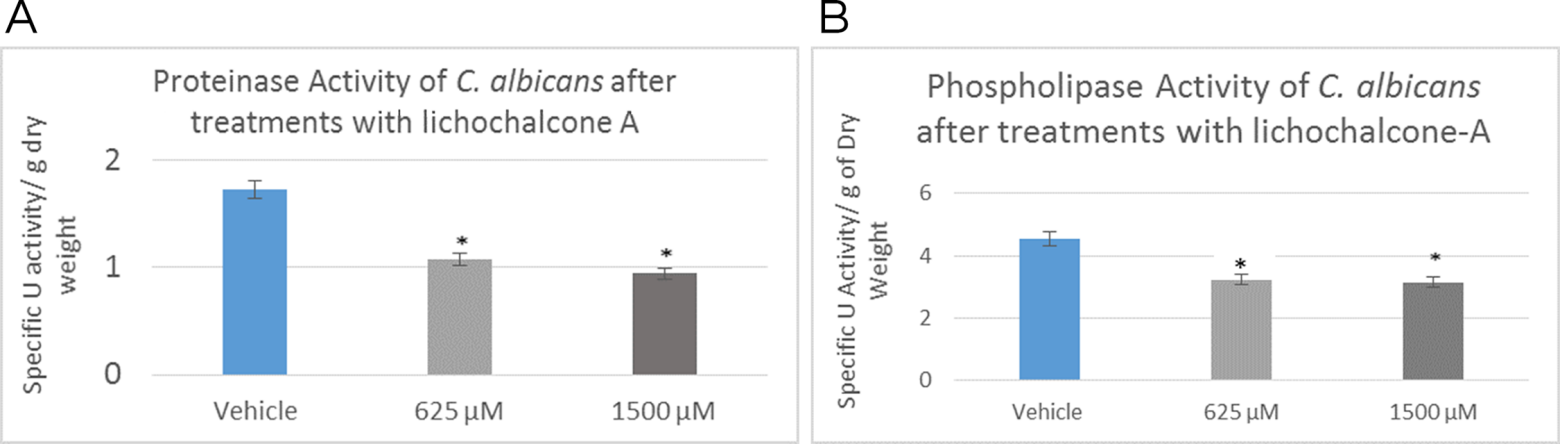
*C*. *albicans* enzymes secretion expressed in U/grams of dry weight after treatment with lichochalcone-A at concentrations of 625 μM and 1500 μM: **A.** Proteinase enzyme activity showed 50% reduction in enzyme activity at 1500 μM concentration; **B.** Phospholipase enzyme activity showed up to 30% decrease in 1500 μM lichochalcone treated biofilms. Significant reduction in proteinases and phospholipases enzyme activities were observed in lichochalcone-A treated biofilms (at concentrations of 625 μM and 1500 μM; equivalent to 10x MIC) in comparison to vehicle control group. *p<0.05.

*C*. *albicans* secreted enzymes were analyzed in the proteinase and phospholipase enzyme assays (Fig 5). Enzyme activity was reported as specific activity unit (measured spectrophotometrically) and normalized by the total dry weight of biofilms (grams). At concentrations of 625 μM and 1500 μM; equivalent to 10x MIC, lichochalcone-A has resulted in significant reduction in enzyme activities of both proteinases and phospholipases (p<0.05).

### ELISA cytokines analysis

**Fig 6:**
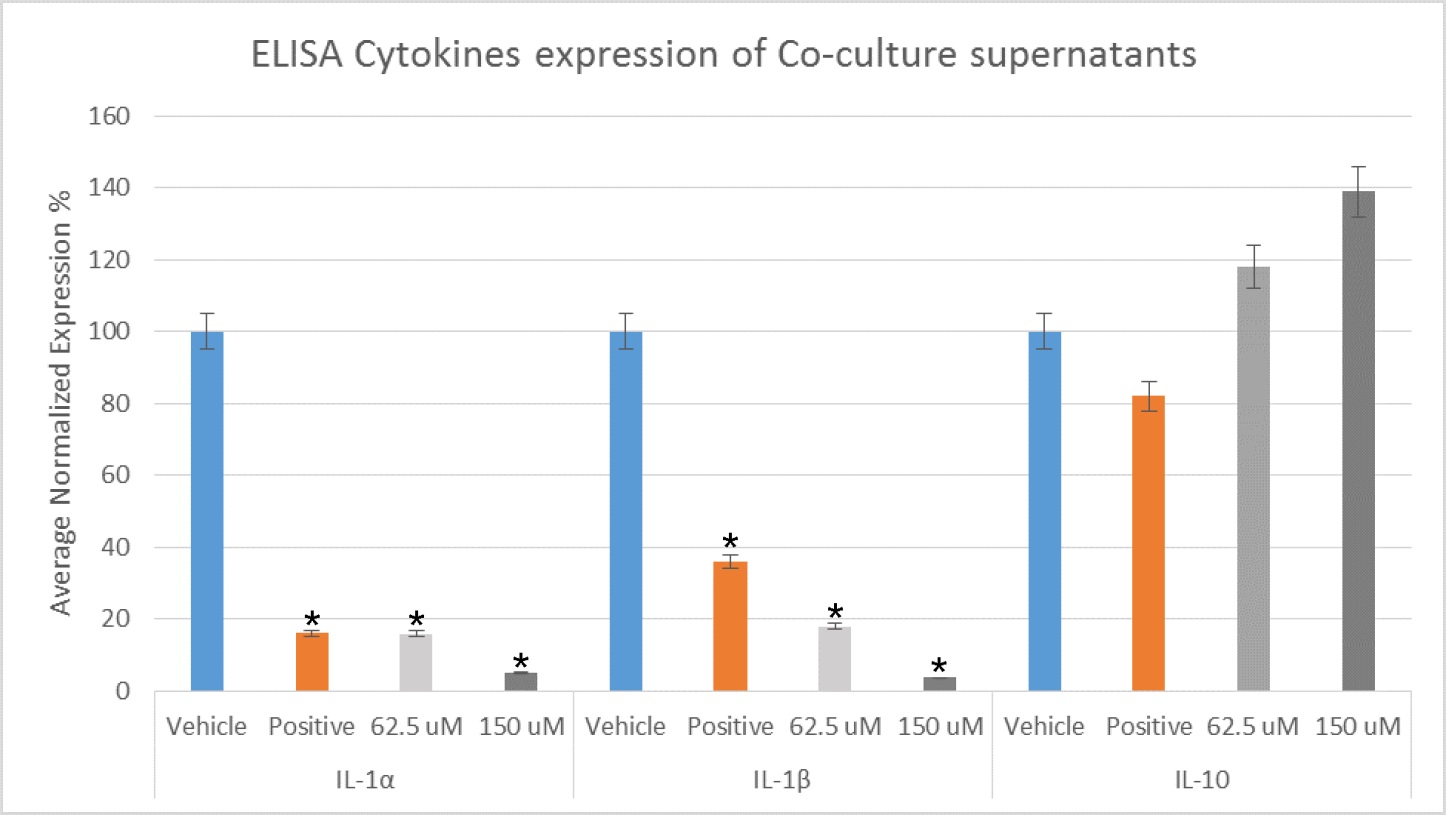
Pro-inflammatory cytokines expression of IL-1α and IL-1β, and anti-inflammatory cytokine expression of IL-10 in *C*. *albicans* treated with lichochalcone-A (62.5 μM and 150 μM), fluconazole (32 μM) (positive control), and 1% ethanol (vehicle control). Significant decrease in IL-1α and IL-1β (*p<0.05), and an increase in IL-10 (results were not statistically significant; p>0.05) in the treated groups compared to the vehicle group. All values are expressed as normalized average expression percentages ± S.e.m (normalized based on the vehicle control groups).

Co-culture *C*. *albicans* biofilms supernatant were assessed for expression of pro-inflammatory IL-1α and IL-1β as well as anti-inflammatory cytokine IL-10, following treatments with lichochalcone-A (62.5 μM and 150 μM). Lichochalcone-A has resulted in a significant reduction in the expression of IL-1α and IL-1β when compared to the vehicle control group (Fig 6), suggesting a modulatory effect of lichochalcone-A on the host pro-inflammatory cytokines expression. On the other hand, the expression of anti-inflammatory IL-10 was increased upon treatment with lichochalcone-A; however, the results were not statistically significant.

### Antifungal activity of lichochalcone-A in a mouse model of oral candidiasis

**Fig 7:**
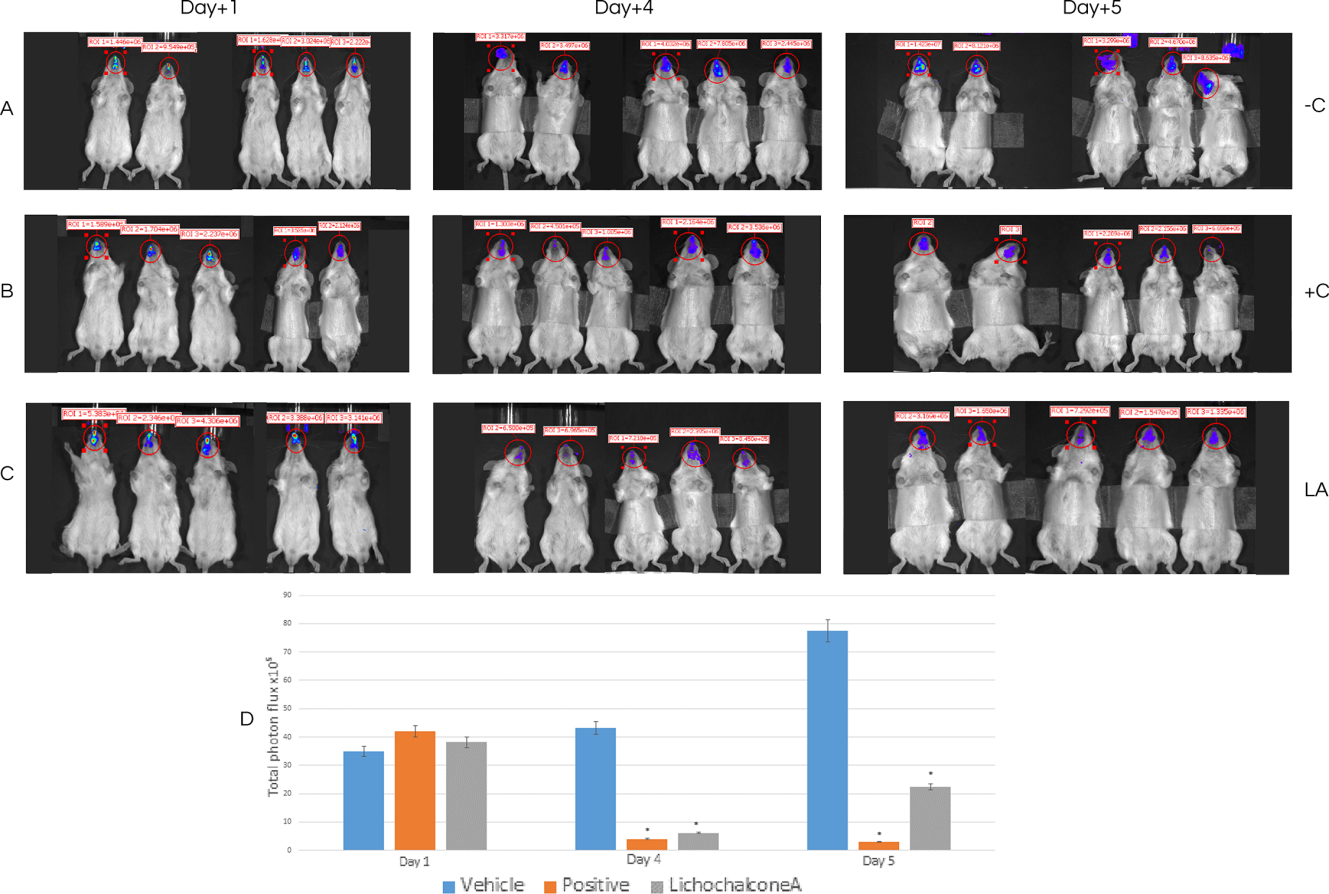
Longitudinal *In vivo* imaging of OC after 1 day, 4 and 5 days post infection with *C*. *albicans* ACTgLUC23 (1x10^7^/ml). **A.** Mice (-C) treated with 1% ethanol as a vehicle control. **B**. Treated (+C) with nystatin, as positive control. **C.** Treated (LA) with lichochalcone-A. **D**. Total photon flux from oral cavities in the images (ROI) of each mouse was quantified with Living ImageR software package. Longitudinal monitoring of fungal load of the mice, grouped as vehicle control, positive control (treated with nystatin), and lichochalcone-A treated group after day 1, 4, and 5 post-infection. Baseline imaging of infection at day 1 post-infection and prior to topical treatments. Significant decrease in total photon flux was observed at days 4 and 5 in lichochalcone-A and nystatin treated groups (*p<0.05).

**Fig 8:**
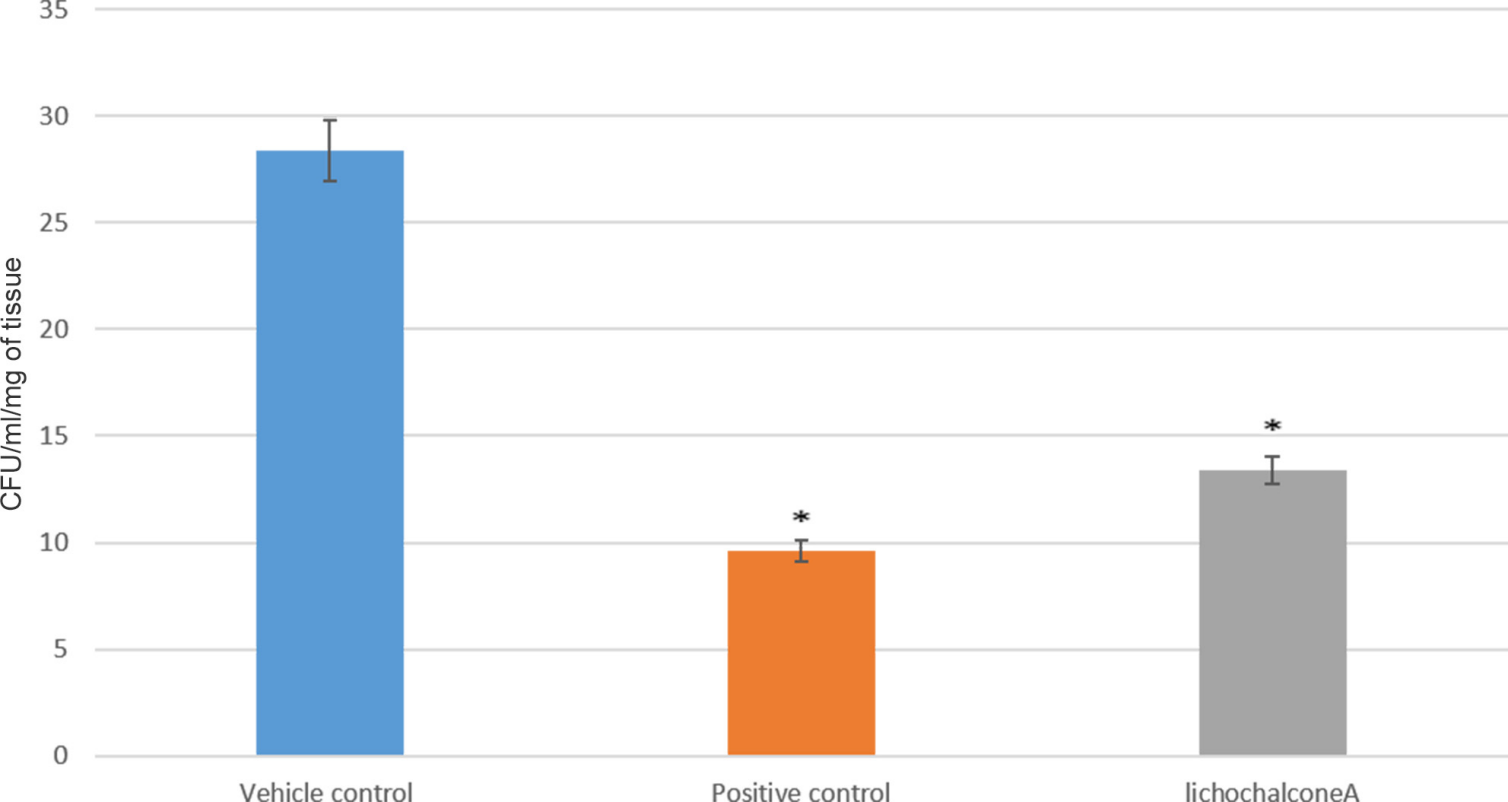
*Ex-vivo* microbiology analysis of tongue samples of mice grouped as vehicle control (1% ethanol), positive control (nystatin treated) and lichochalcone-A treated group. Fungal load of the tongues are expressed as colony formation unit (CFU)/ml/mg of tissue. Lichochalcone-A group showed more than 50% reduction in colony count compared to vehicle control group (*p<0.05).

**Fig 9:**
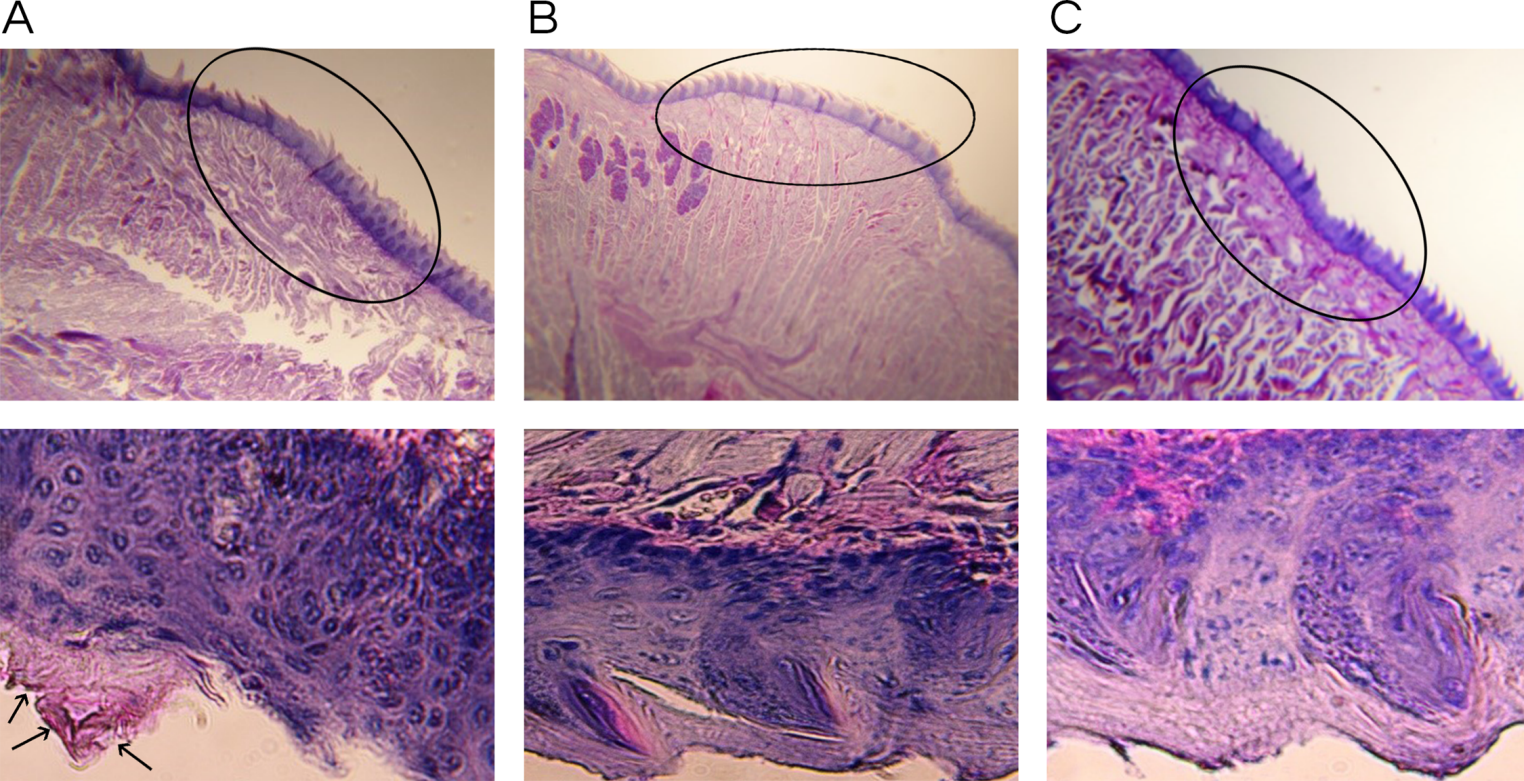
**Histopathology of tongue sections at 4x magnification (top) and (400x magnification) bottom from** A. Vehicle control, B. Positive control treated with nystatin, and C. Lichochalcone-A. Circles on top images shows a section of the epithelium layer that is enlarged below at 400x magnification. Tongue section (A, left panel) from vehicle control showed yeasts and hyphae (arrows) invasion of the dorsal papillary architecture. Presence of *C*. *albicans* is indicated by arrows pointing to pink staining (A, lower left). Tongue sections from panel B, positive control, and panel C, lichochalcone-A, did not show signs of fungal burden.

The antifungal effects of lichochalcone-A in comparison to nystatin (100 mM) as a positive control and 1% ethanol as a vehicle control were assessed in a mouse model of oral candidiasis established by inoculating a bioluminescent *C*. *albicans* (*SKCa23-ActgLUC*) (1 x 10^7^ CFUs/ml) in the oral cavities of the mice. A baseline IVIS imaging was obtained at day one post-infection to confirm that all the mice were infected and to quantify the initial fungal load in the oral cavities. Topical treatments at 30 seconds intervals were given to the mice twice daily for a duration of 5 days. Longitudinal monitoring of imaging of the oral fungal infection was achieved by pipetting coelenterazine substrate into the oral cavities of the mice at days 4 and 5 post-infection. It was observed that at days 4 and 5 of imaging, there was a significant reduction (p<0.05) of the total photon flux in the lichochalcone-A and the nystatin treated groups in comparison to the vehicle control, in which total photon flux continued to increase over the 5 days imaging period (Fig 7). Immediately after euthanasia (day 6 post-infection), the tongues were sectioned longitudinally for microbiology and histopathology analysis of the fungal load. Tissue samples collected for the microbiology analysis were weighed to normalize data of CFU/ml and serial dilutions in PBS were performed prior to plating on sabouraud agar plates. Consistent with the results obtained from the photon flux emission analysis, there was a significant reduction (p<0.05) of CFU/ml/ mg of tissue in the lichochalcone-A and nystatin groups in comparison to the vehicle control (Fig 8). Moreover, tongue samples of the lichochalcone-A and nystatin treated groups displayed less severity of hyphal invasion in comparison to the vehicle control, as noted in PAS-stained tongue sections (Fig 9).

## Discussion

Natural compounds, such as polyphenols, possess potential therapeutic characteristics against fungal infections due to their readily bioavailability and their known antimicrobial activities [39]. Lichochalcone-A is a polyphenol naturally found in licorice root (*Radix Glycyrrhizae*) root, which has been known for its broad bioactivities, such as anti-inflammatory, anti-tumor, and antimicrobial effects [40]. The resistance of *Candida* species to conventionally used antifungal agents, such as triazoles, represents a major challenge for the treatment of candidiasis, especially in individuals with diminished immune response; for example, in HIV patients. Lichochalcone-A presents as a potential antimicrobial agent, as it has been incorporated in traditional Asian remedies for thousands of years [17]. However, scientific validation of its antifungal effects as well as an understanding of how lichochalcone-A may modulate virulence factors of *C*. *albicans* are necessary to establish its safety for therapeutic purposes.

In our *in vitro* study, we tested the antifungal activity of lichochalcone-A against *C*. *albicans* biofilms. Susceptibility assays of lichochalcone-A showed inhibition of fungal growth of *C*. *albicans* MYA2876 and ATCC 90028 at a minimum inhibitory concentration (MIC) of 62.5–150 μM and 62.5–100 μM, respectively (Table 1). The minimum fungicidal concentration (MFC) of lichochalcone-A against *C*. *albicans* (100–150 μM) was similar to the MFC of fluconazole (90–100 μM, which has been considered to be a gold standard of antifungal agent. In addition, lichochalcone-A was potent against the fluconazole-resistant strain 321182 at a relatively lower MIC and MFC concentrations of 65 μM and 150 μM, respectively, in comparison the fluconazole MIC and MFC values of 100 μM and 350 μM, respectively. In the biofilm assay model using *C*. *albicans* MYA2876, 10x the concentrations of MIC (625 μM) were used due to the tenacious nature of biofilms to eradicate. Biofilms treated with lichochalcone-A showed significant (p<0.05) reduction in fungal viability, as illustrated by the decrease in CFU/ml/grams of dry weight of biofilm sample in comparison to the control groups (Fig 3).

Using a co-culture model, we were able to assess qualitatively the distribution of immature *C*. *albicans* biofilms, primarily composed of blastopores and a few hyphae, in conjunction with fibroblasts in response to lichochalcone-A and the respective controls. It was found that lichochalcone-A (62.5 μM) had a substantial decrease in *Candida* formation without altering the fibroblast distribution or increasing the fibroblast dead cells distribution, which was comparable to the positive control group (Fig 4). Thus, the co-culture model has provided insights about a complex system of host cells interacting with *Candida* as well as the tested compounds, showing a strong antifungal effect with minimal toxicity. In addition, co-culture supernatant were assessed for expression of host pro-inflammatory and anti-inflammatory cytokines. Lichochalcone-A was found to significantly decrease the expression of pro-inflammatory cytokines IL-1α and IL-1β (Fig 6), suggesting it may have a modulatory role on the host pro-inflammatory response to help eradicate fungal infections [41].

One of the goals in this *in vitro* study was to investigate how lichochalcone-A may alter critical virulence factors that contribute to the pathogenicity of *C*. *albicans*. Proteolytic enzymes, such as proteinases and phospholipases, are enzymes secreted by *C*. *albicans* and are often associated with tissue degradation, hyphal formation, and host invasion, which are critical factors linked to the pathogenicity of *C*. *albicans* [12,13]. *C*. *albicans* secreted aspartyl proteinases (SAPs) have been reported to elicit a destructive effect on the host tissue during mucosal infections, as they facilitate hyphal invasion and activate the degradation of E-cadherin, a major protein present in epithelial cell junction [12,13]. It has been noted in the literature that *C*. *albicans* secreted aspartyl proteinases are often associated with virulence factors contributing to the progression of candidiasis [42]. In fact, during fungal infections, there is generally a higher gene expression of SAPs, which is often associated with hyphal formation and the induction of rim101p, a transcription factor that mediates the degradation of E-cadherin protein of the epithelial cell junction [13]. Similarly, phospholipases B1, B2, C and D of *C*. *albicans* play a significant role in the invasion of the host tissue, as noted by their high gene expression during fungal infection [43]. More specifically, phospholipase B (PLB) proteins were shown to have hydrolytic activity, as they hydrolyze acyl ester bonds in phospholipids and lysophospholipids and catalyze lysophospholipase-transacylase reactions [44]. It was determined that the PLB multigene family of the opportunistic fungal pathogen *C*. *albicans* encodes for CaPLB5, a putative secretory protein with a predicted GPI-anchor attachment site. The ability of *C*. *albicans* to attach itself to the host tissue is considered a key pathogenic characteristic and hence, genes encoding for attachment proteins, such as PLB, may be potential virulence determinants [44]. In our study, proteinase and phospholipase enzymatic activities were investigated using 625 μM and 1500 μM concentrations of lichochalcone-A (Fig 5). It was found that these concentrations of lichohalcone-A significantly decreased the enzyme activities of both proteinases and phospholipases, which suggests that one possible antifungal mechanism of action of lichochalcone-A involves the inhibition of the protease secretions.

In our *in vivo* study on mouse model, we used a bioluminescent strain of *C*. *albicans* (*SKCa23-ActgLUC*) in combination with coleranterazine substrate to allow for longitudinal imaging of oral candidiasis infections of mice [19]. Under immunosuppression with cortisone acetate, mice were rendered susceptible to fungal infection as long as immunosuppression was maintained [35]. We showed that oral topical treatments of lichochalcone-A (7.5 mM) significantly reduced the fungal load of the oral candidiasis in mouse model over a period of 5 days. At day 4-post infection, the efficacy of lichochalcone-A in reducing the total photon flux was comparable to nystatin, the most commonly used antifungal agent for oral candidiasis (Fig 7) [45]. At day 5 post-infection, there was a slight increase of the total photon emission in the lichochalcone-A treated group. However, the results of the photon flux for lichochalcone-A group were similar to the nystatin group at day 5, both of which were significantly lower than the vehicle control group. A possible explanation for the slight increase in photon flux at day 5 for the lichochalcone-A group was possibly due to the prolonged duration of the animals’ immunosuppression and their overall weaker immunity over time, thus, rendering them susceptible to dissemination of infection progressively. However, the microbiology analysis of the *ex-vivo* tongue samples confirmed the overall efficacy of lichochalcone-A as a potent antifungal agent, as there was a significant reduction in CFU/ml/ mg of tongue tissue in the lichochalcone-A treated group in comparison to the vehicle control group (Fig 8). Furthermore, the histopathology of the tongue indicated a much less severity of *candida* lesions and hyphal invasion in the lichochalcone-A treated group when compared to the vehicle control group (Fig 9). On the other hand, no tissue necrosis was observed in the lichochalcone-A treated tongue sample, suggesting low toxicity to *in vivo* cells.

In conclusion, the findings of the present study support lichochalcone-A as a promising antifungal natural compound, as demonstrated using *in vitro* and *in vivo* assays. Future direction of research may evaluate the pharmacodynamics of lichochalcone-A in an animal model of OC to assess the ideal concentration necessary to attain optimal antifungal effects. Furthermore, the duration of the oral topical treatment may also be studied in future research. Ultimately, lichochalcone-A presents as a potential antifungal agent, which may be investigated in further studies for the treatment and/ or the prevention of oral candidiasis in clinical settings.

## Supporting Information

S1 Dataset Raw data of *in* vitro, in *vivo* antifungal biofilm assays of lichochalcone-A against *C*. *albicans* (MYA 2876) and *C*. *albicans* (*SKCa23-ActgLUC*), including time-kill assays, proteinases and phospholipases enzymatic activities, and cytokines expression of the host fibroblast cells. (XLSX)
